# Muscle Strength, Physical Fitness, Balance, and Walking Ability at Risk of Fall for Prefrail Older People

**DOI:** 10.1155/2022/4581126

**Published:** 2022-12-07

**Authors:** Yan-Cheng Lee, Shu-Fang Chang, Ching-Yun Kao, Hsiao Chi Tsai

**Affiliations:** ^1^School of Nursing, College of Nursing, National Taipei University of Nursing and Health Sciences, Taipei, Taiwan; ^2^Saint Paul's Hospital, No. 123, Jianxin Street, Taoyuan District, Taoyuan City, Taiwan; ^3^Cardinal Tien Hospital, No. 15, Chezi Rd., Xindian Dist., New Taipei City, 23155, Taiwan

## Abstract

**Aim:**

This study was to explore the relationship of older adults' demographic information, physiological indices, and stages of frailty with their risk of falling.

**Methods:**

In the cross-sectional study, a total of 221 older adults with the mean age 74.9 (SD = 6.8) years old were surveyed by senior fitness test.

**Results:**

Results were observed in terms of participants' physical fitness, with significant differences being observed in the correlations of left-hand grip strength (*t* = 5.05, *p* < .000), right-hand grip strength (*t* = 6.03, *p* < .000), and total grip strength (*t* = 5.70, *p* < .000), time up and go test (*t* = −6.25, *p* < .000), and 30-sec chair stand test (*t* = 7.19, *p* < .000) with the risk of falling. According to the logistic regression analysis results, long-term medication (OR = 0.12, 95% CI =0.02-0.62, *p* < .01) and right-hand grip strength (OR = 0.86, 95% CI =0.76-0.97, *p* < .01) are the main predictors of older adults' risk of falling.

**Conclusions:**

Older females with low education, history of falls, weaker grip strengths; taking longer to finish the TUG test; and standing fewer times during the 30-second chair stand test were at risk of fall. In prediction, older people using long-term medication were at lower risk of falling, and the greater the hand grip strength was, the lower the fall risk was. According to the research results, nursing personnel must develop care programs and improve older adults' risk of falls.

## 1. Introduction

Population aging is a worldwide crisis deserving attention. According to the World Health Organization, an “aging society” comprises more than 7% people aged 65 years or older; for an “aged society”, the corresponding ratio exceeds14%; and for a “hyper-aged society”, the ratio exceeds 20%.The number of people aged 65 years or older worldwide is estimated to rapidly rise from 900 million to 3200 million from 2016 to 2100. According to statistics from the Ministry of the Interior, R.O.C. (Taiwan), the ratio of adults aged 65 years or older in Taiwan reached10.6% by the end of 2009. This was estimated to rise to 14.7% in the next 10years and further to 37.5% in 2056 [1].

Research on frailty indicated the prevalence of frailty among older adults in the United States was 9.6%, and the prevalence of prefrailty was 47% [2]. In the U.K., the prevalence of frailty among older adults was 14% [3]; the corresponding prevalence in Europe was 2.6%; and the prevalence of prefrailty in Europe was 38.8% [4]. According to the study by Biritwum et al. [5], the ratio of adults aged 50 years or older in six countries—China, Ghana, India, Mexico, Russia, and South Africa Republic—accounted for 43% of the adults in this age group worldwide. Investigations showed the prevalence of frailty in older adults to be the lowest in China (13.1%) and highest in India (55.5%). The result of the investigation by Yu et al. [6] on the frailty prevalence among older adults in rural areas and cities in Taiwan and Hong Kong demonstrated that frailty prevalence in rural areas in Taiwan was 38.10% and in Taiwanese cities was 33.06%. Frailty prevalence in Hong Kong was 16.57%.

According to research reports, the prevalence of falls among community-dwelling older adults was approximately 30% to 40%, and half of the older adults suffered from recurrent falls [7]. According to the research report of the “Taiwan Longitudinal Study of Aging” in Taiwan in 2015, the incidence of falls in the past year among community-dwelling older adults aged 65 years or older was 20.7%, and 37% of these adults fell recurrently [8]. In Taiwan, the mean annual hospitalization expense due to falls was between NT$90,000 to NT$130,000 per older adult. For older adults suffering from hipbone fractures due to falls, the annual medical expenses searches approximately NT$3,000,000,000 [8, 9]. Research in the Asian region that has investigated community-dwelling older adults in Taiwan indicated that frailty prevalence among people aged 65 years or older was approximately 14.1%, prefrailty prevalence was 53.7%, and the prevalence of both increased with age [10], meaning that frailty care and prevention are crucial. According to the Department of Health, Education, and Welfare of the United States, the estimated annual medical expense due to frailty was between US$11,800 and US$ 26,200 million [11]; effective frailty prevention could reduce high medical costs. Therefore, assessing the relevant risk factors early on, before frailty or during prefrailty, helps to prevent future adverse health incidents such as falls.

Therefore, this study conducted investigations in communities to examine community-dwelling older adults' basic attributes, physical fitness, and frailty stages in relation to fall risk predictability. The results showed that it was possible to discover fall risk factors early enough to prevent or postpone the future occurrence of adverse health incidents among older adults, subsequently alleviating family care load and improving quality of life for older adults.

### 1.1. Aims

This study first analyzed the basic attributes, physical fitness, frailty stage, and fall risk among community-dwelling older adults, and this was followed by an analysis of differences in basic attributes, physical fitness, frailty stage, and fall risk. Last, the study explored participants' basic attributes, physical fitness, and frailty stages in relation to fall risk predictability.

### 1.2. Literature Review

#### 1.2.1. Frailty

Frailty, a continuum of malfunction in physiological systems, is a complex and dynamic condition in which elements related to the body, mind, and society interact. It is related to age but preventable and predictable by certain factors; therefore, aging does not necessarily cause frailty. Meanwhile, frailty is not related to any specific disease but increases comorbidities [10]. According to Fried et al. [12], frailty signifies a progressive physiological decline in several systems of the body, leading to the risk of adverse health outcomes such as physiological disability, loss of physiological reserve, and increased incidence of mortality, falls, disability, and hospitalization. The more commonly applied frailty assessment indexes are the five indicators of shrinking, low grip strength, exhaustion, slowness, and low activity level proposed by Fried who established three frailty stages: nonfrail, prefrail, and frail [12].

#### 1.2.2. Fried Phenotype of Frailty Derived from Cardiovascular Health Study (CHS)

Fried et al. [12] distinguished five major symptoms of frailty: (1) shrinking, (2) low grip strength, (3) exhaustion, (4) slowness, and (5) low physical activity level. Grip strength and 15-feet walking require physical fitness tests. Individuals who meet three of the five criteria are identified as physiologically frail, those who meet one or two criteria are classified as physiologically prefrail, and those meeting none of the five criteria are classified as physiologically nonfrail.

#### 1.2.3. Study of Osteoporotic Fracture Index (SOF Index)

The criteria for frailty of Study of Osteoporotic Fracture (SOF) Index proposed by Ensrud et al. [13] comprised three indexes corresponding to the following questions: (1) have you experienced an unintentional weight loss of ≥3 kg or 5% during the past year? (2) Can you do five chair stands without using your arms? (3) Do you feel full of energy? Respondents who answered “no” to one of the questions were deemed prefrail, and those who answered “no” to two or more questions were deemed frail.

The aforementioned literature showed that CHS index and SOF index were equally effective methods for assessing the risk predictability of negative care outcomes in community-dwelling older adults, such as recurrent falls, fractures, disability, hospitalization, and mortality. Both were applicable to community-dwelling older adults and amply discriminating. However, the CHS index was less applicable to the community context because it comprised more questions and required more complex tests (grip strength and walking speed tests) than the SOF index did [14].Osteoporotic fractures studied by Ensrud et al. [13] indicated that the relatively few questions and an easy measurement method of the SOF Index made it as effective as the CHS Index in terms of risk predictability for negative care outcomes of community-dwelling older adults, including recurrent falls, disability, fractures, hospitalization, and mortality; the measurements were also easier to apply clinically thanks to the simple test procedures [15]. Therefore, the SOF Index was applied in this study.

#### 1.2.4. Studies on Frailty and Falls

Research indicated that frail older adults are less resistant to pressure when experiencing stressful conditions such as injury, infection, anesthesia, surgery, and medication and that they experience recurring adverse health conditions, including illness, falls, disability, hospitalization, stays in long-term care institutions, and mortality [10]. Lu et al. [16] conducted frailty assessments of 189 people aged 65 years or older using Fried indexes and cross-sectional research in outpatient clinics for chronic diseases. Among the 19% of the prefrail patients, falls over the preceding year, memory problems, dysphagia, fecal incontinence, pain, balance problems, and constipation were significantly more recurrent. Prefrailty-related predictors included five or more chronic diseases (OR = 3.99, 95% CI =1.26–12.60), constipation (OR = 5.32, 95% CI =1.99–14.38), falls over the preceding year (OR = 3.15, 95% CI =1.07–9.22), disability in action (OR = 14.03, 95% CI =3.75–52.56) and use of eight or more medicines for chronic diseases (OR = 4.19, 95% CI =1.13–15.54). Tseng [17] used a retrospective longitudinal study on 341institutionalized adults aged 65 years or older in which the proportion of prefrail study participants was 53.7%, and the proportion of recurrent falls increased with frailty level. An analysis of the relationship of adverse health outcomes indicated that the chance of falls within two years in prefrail older adults with low walking speeds was 2.77 times that for healthy older adults (OR = 2.77, 95% CI =1.20–6.41, *p* = 0.018). Furthermore, results from the observation by Cigolle et al. [18] of 11,093 older adults aged 65 years or older in nursing homes indicated that the frailty prevalence was proportional to age. The study by Muir et al. [19] on 210 institutionalized prefrail older adults (70% men and 30%women; mean age =79.9years old, SD =4.7) demonstrated that the fall risk for those with inferior balance was 1.5 to 1.6 times higher. The study by Gonzalez-Vaca [20] on 331 institutionalized adults aged 65 years or older showed that 31.2% of the study participants were nonfrail and prefrail older adults; those who had fallen during the previous six months had significantly higher frailty levels in comparison with those who had not.

According to the aforementioned literature, older adults' physical fitness for muscle strength, balance, and walking ability, for example, was closely related to the frequency of falls. Literature on falling was mostly set in institutions and outpatient departments of hospitals. Few research in Taiwan on prefrail community-dwelling older adults was based on objective measurements that involve investigating older adults' muscle strength, physical fitness, balance, and walking ability and how these factors influenced the incidence of falls. Therefore, this study explored, through community investigation, prefrail community-dwelling older adults' basic attributes, physical fitness, and frailty stages in relation to fall risk predictability.

## 2. Method

### 2.1. Design

This study applied cross-sectional research design, purposive sampling, and structured questionnaires to collect and investigate basic attributes, physical fitness, and frailty stages in relation to fall risk predictability for community-dwelling adults aged 65 years or older.

### 2.2. Research Participants

This study collected data on prefrail community-dwelling adults aged 65 years or older in northern Taiwan. Older adults fulfilling the following criteria were included in the study: (1) an age of 65 years or older without being banned from doing exercise, (2) clear consciousness and ability to communicate in Mandarin or Taiwanese, (3) prefrail status established through SOF Index screening, and (4) willingness to participate in the study and ability to complete questions independently or with assistance from researchers on site. The following criteria were used for exclusion:(1) inability to participate in the study due to serious visual and audio impairments, (2) balance problems and inability to participate in the tests (for example, inability to sit, stand, or walk), and (3) with wrist fractures.

### 2.3. Sample Size

This study used G-Power 3.1software: *α* was set as 0.05, and Power was set as 0.9 with 95% of the confidence level and 5% of the confidence interval. Sample size was calculated; the total sample size was at least 221. The sex ratio of male to female in the assessable population is 1 : 2. Therefore, for 221 sampling participants, 75 males and 146 females are expected to be collected.

## 3. Research Tool

### 3.1. Demographic Characteristic

The characteristic included age, gender, marital status, living arrangement, education level, alcohol use history, history of falls, medical history, long-term medication, exercise status, and insomnia status.

### 3.2. Physical Fitness

#### 3.2.1. Body Mass Index

Overweight and obesity mean being abnormally or excessively fat and may lead to health risks. The main measurement is body mass index (BMI), which is obtained by the body weight(kg) divided by the square of the body height (m^2^). The scores proposed by the WHO were used(18.5-25.0 kg/m^2^: normal, 25.1–26.9 kg/m^2^: overweight, ≥27 kg/m^2^: obese). Chronic diseases such as cardiovascular diseases, diabetes, and cancer were caused by overweight and obesity [8]. BMI was also relevant to frailty, with abnormal weight (underweight or overweight) associated with a higher risk for frailty [21].

#### 3.2.2. Grip Strength

The grip strength test in this study referred to the senior fitness test developed by Rikli and Jones [22]. They developed and validated a functional fitness test for community-residing older adults. Participants used both hands in turn to grip the gripper twice, and the maximum value was recorded as the grip strength value. This tests muscles of the upper extremity by measuring the maximum muscle strength in static contraction with a digital dynamometer. The subject is asked to sit down with the elbow joints at an angle of 90° and with the knuckles gripping a digital dynamometer with the greatest possible force continuously for two seconds. The overall grip strength is the sum of the left and right handgrip strengths; the measurement unit is kilograms. The intra-class correlation coefficient (ICC) of the test was 0.81 [22], and the content validities for men and women were 0.81 and 0.78, respectively [23]. The test–retest reliability between the left and right hands of 21 healthy older adults by Bohannon and Schaubert [24] using a Jamar dynamometer indicated no significant difference. Intraclass correlation coefficients for the left and right hands were 0.954 and 0.912, respectively.

#### 3.2.3. Timed “Up and Go” Test

The TUG test in this study referred to the senior physical fitness test developed by Rikli and Jones [22]. The participant stands up from a seated position upon hearing the “go” command and walks to the point 2.44 meters in the front of them before turning around and walking back to the front of the chair; the timer stops when the individual turns around and sits down. The test participant repeats the test twice, and the faster result is recorded. The shorter the respective time, the better the dynamic balance of the individual is considered [22].

According to Shumway-Cook et al. [25], when the community-dwelling older adults' cutoff point was 13.5 seconds, their sensitivity (87%) and specificity (87%) in terms of fall prediction were high. Several past studies have verified that the TUG test exhibited high intrarater reliability(ICC =0.95–0.99) and interrater reliability (ICC =0.56–0.98) [26, 27]. Regarding validity, the results demonstrated that the TUG test was moderately to highly correlated with the Berg Balance Scale (BBS) (*r* =0.47–0.74) in testing community-dwelling older adults [26, 28].

#### 3.2.4. 30-Second Chair Stand Test

The test uses an armless chair with a fixed height (43–46 cm).The test participant sits in the middle of the chair without leaning on the chair back. During the test, the participant places each of their hands on the opposite shoulders, crossed at the wrists, while keeping the feet flat on the floor. The participants rise to a full standing position upon hearing “go” and then sit down again, thus completing the cycle. The number of times the participants are able to complete in 30 seconds were recorded. The tools include a stopwatch and an armless chair. The test takers are given one chance, and the number of times is used as the measurement unit [22].

Jones et al. [29] divided 76 community dwellers (34 men and42 women) into three age groups and compared the number of times participants of each group were able to stand in 30 seconds. The results indicated a favorable test–retest reliability for 30-second chair stands (0.84 < *R* < 0.92, *p* < .05), and the number of times participants were able to stand decreased with age (*F* = 4.4, *p* < 0.01); a correlation was observed between the number of times older adults were able to stand and leg muscle strength adjusted by weight (*r* = 0 .77, 95% CI = 0.64–0.85). This indicated that older adults' leg muscle strength and endurance were significantly correlated to their activity levels as well as to future falls and hospitalization, which was also highly discriminating.

#### 3.2.5. Frailty Stages

The SOF Index of Ensrud et al. [13] indicated that its relatively few questions and ease of measurement made it as effective as the CHS Index in terms of risk predictability of negative care outcomes in community-dwelling older adults, including for recurrent falls, disability, fractures, hospitalization, and mortality; the measurement was also easier to apply clinically thanks to the simplicity of its test procedures [15, 30]. Therefore, the SOF Index [13] was applied as the criteria for this study. It comprised three indexes corresponding to the following questions: (1) have you experienced an unintentional weight loss of ≥3 kg or 5% during the past year? (2) Can you do five chair stands without using your arms? (3) Do you feel full of energy? Respondents who answered “no” to one of the questions were deemed prefrail, and those who answered “no” to two or more questions were deemed frail.

#### 3.2.6. Falls

This study used the BBS for measuring a person's dynamic balance ability, which takes only15 to 20 minutes. It includes tests on 14 daily tasks, with the score for each ranging from 0 to 4. The total score is 56; test participants scoring45 or more are deemed to possess good balance ability and the ability to walk independently, whereas those scoring under 45 are deemed to be inferior in terms of physical balance and to be at risk of falls. According to Berg et al. [31] and Chou et al. [32], the Cronbach's alpha of this scale was 0.97–0.98 and 0.98, respectively. The ICC of the BBS was 0.93(95% CI: 0.87–0.96), demonstrating a relatively high internal consistency [33].

#### 3.2.7. Research Ethics

The researchers first determined the principle investigator and submitted the project to the Research Ethics Committee of the National Taiwan University for review (case number obtained after approval: 201903ES021) prior to execution.

#### 3.2.8. Data Analysis

This study used SPSS Statistics 21.0 software for Windows to organize and analyze the data. Status of basic attributes, physical fitness, and fall risk were expressed as *n* (%) and mean ± SD. Differences of the basic attributes, physical fitness, and fall risk were analyzed using chi-square test (*χ*^2^) and independent sample *t*-test. Risk predictability of fall risk on basic attributes and physical fitness was used by linear regression analysis.

## 4. Results

### 4.1. Basic Characteristic, Physical Fitness, Frailty Stages, and Fall Risk of Community-Dwelling Older Adults

The mean age of the study participants was 74.9 (SD = 6.8) years old. The majority of the older adults enrolled were between 65 and 75years of age (52.9%), were women (146 women,66.1%), were married (167persons, 75.6%), and lived with family (191persons, 86.4%). The highest education level of the majority of participants was graduation from elementary school (121persons, 54.8%), and the next most common maximum education level was illiteracy (61persons, 27.6%). The majority of participants never drank alcohol (165persons, 74.7%). The number of those who had fallen was 49 (22.2%), with the majority having fallen only once. The majority of participants were not using long-term medication (185 persons, 83.7%), and the average number of medicines used on a long-term basis by these adults was 2.11, with hypertension representing the major disease being treated (*n* = 113; 28.5%). The majority of participants exercised “more than three days per week” (191 persons, 86.4%). The majority (59.7%) of participants were not insomniacs (Table 1).

The mean BMI of the study participants was 25.18 kg/m^2^. The majority was “overweight” (83 persons, 37.6%). The mean left-handgrip strength was 25.22 kg (SD = 8.11), and the mean right-hand grip strength was 26.31 kg (SD = 8.66). The mean overall grip strength reached 51.54 kg (SD = 16.41). The mean for the TUG test was 8.12 seconds (SD = 3.04). In the 30-second chair stand test, the mean was 16.41 times (SD = 5.02) (Table 1).

### 4.2. Analysis of the Differences in Older Adults' Basic Characteristic, Physical Fitness, Frailty Stages, and Fall Risks

In terms of basic attributes, age (*t* = −7.42, *p* < .000), gender (*χ*2 = 3.96, *p* < .04), education level (X^2^ = 32.28, p < .000), fall history (X^2^ = 8.95, p < .03), the use of long-term medication (*χ*2 = 14.79, *p* < .000), and the number of long-term medicines used (*t* = −.79, *p* < .000), and the number of at risk of falls and among the older adults not at risk were significantly different(*p* < .05). Regarding the physical fitness, significant differences (*p* < .05) were observed between the left hand grip strength (*t* = 5.05, *p* < .000), right handgrip strength(*t* = 6.03, *p* < .000), overall grip strength(*t* = 5.70, *p* < .000), the TUG test result (*t* = −6.25, *p* < .000), the TUG test result (*t* = 7.19, *p* < .000) of study participants at risk of falls compared with for study participants not at risk of falls. In terms of frailty stage, a significant difference (*p* < .05) was observed between the older adults at risk of falls and those not at risk (Table 2).

### 4.3. Older Adults' Basic Characteristic, Physical Fitness and Frailty Stages in relation to Fall Risk Predictability

This study demonstrated significant differences between use of long-term medication (OR = 0.12, 95% CI =0.02–0.62, *p* < .01) and right handgrip strength (OR =0.86, 95% CI =0.76–0.97, *p* < .01) for older adults at risk of falls compared with for older adults no at risk. A further analysis demonstrated that older adults not using long-term medication were at lower risk of falling than those using long-term medication were. Greater right handgrip strength was associated with lower risk of falling (Table 3).

## 5. Discussion

### 5.1. Community-Dwelling Older Adults' Basic Characteristic, Physical Fitness, FrailtyStages, and Fall Risk

The mean age was 74.9 years old, with women and married people comprising the majority. Among those using long-term medication, the average number of medicines used was 2.11, with hypertension constituting the major disease being treated. These findings were similar to those of a domestic study [34] on community-dwelling older adults regarding the correlation of health conditions and physical function with falls. Another related study was Chen [35] on the factors affecting community-dwelling older adults' fear of falling: The majority had fallen once. In addition, the results of this study resembled those of Ko [36], on the prevalence of and risk factors for falls among older adults in Taiwan, which found the medical history included bone and joint diseases, hypertension, and cataract. Other similar results came from Lin [37] on fall prevention and related factors for seniors in Taipei community care centers; the exercise status of the study participants was also “more than three days per week.” In addition, an investigation by scholars on the cumulative incidence of falls and related factors for older adults in Shipai, Taipei, yielded a similar result as follows: the majority was not insomniacs [38].

According to the World Health Organization (WHO), the BMI of reference for older adults must be normal between 18.5 and 25. In this study, the BMI groupings showed the majority to be close to overweight, and the respective result was close to that of Lee [39] on factors related to falls among community-dwelling older adults, which yielded a mean BMI 25.9. In this study, the mean time for the TUG test was 8.12 seconds, and the mean number of chair stands achieved in the 30-second chair stand test was 16.41. These values are superior to the results of tests by domestic scholars on community-dwelling older adults: The meantime for the TUG test was 10.86 seconds, whereas the mean number of stands achieved in the 30-second chair stand test was 13.39 [40]. The TUG test by Chen [34] on community-dwelling older adults yielded a meantime of 12.48 seconds, and the mean number of stands achieved in a 30-second chair stand test was 10.89, both of which results were inferior to this study's. According to the results of physical fitness tests conducted by scholars on community-dwelling older adults, the TUG and 30-secondschair stand test results for frail older adults were inferior to those for nonfrail and prefrail adults [41, 42].

Prefrail older adults accounted for 34.4% of our study's participants, a proportion close to those in prefrailty in related studies. Investigations on the five frailty indexes and adverse health outcomes in community-dwelling older adults by domestic scholars found the prefrailty prevalence to be 32.3%, a result close to that of this study [17]. The investigation by Wei [43] on the predictability of community-dwelling older adults' frailty and quality of life found the prefrailty prevalence to be 37.4%, which was also close to the result of this study. Domestic scholars' research on the prevalence of and factors related to prefrailty among older adults in Taiwan found the prefrailty prevalence to be 30.6%, a result close to that of this study [44]. Moreover, the participants of this study were screened using the BBS. The results showed that 39.8% of adults aged 65 years or older exhibited fall risk, and this resembled the results of other studies demonstrating that the incidence of falls increased with age, and the annual incidence of falls in community-dwelling adults aged 65 years or older was 28%–35%, whereas that of adults aged 75 years or older rose to 32%–42% [45].

### 5.2. Difference Analysis of Community-Dwelling Older Adults' Basic Characteristic, Physical Fitness, Frailty Stages, and Fall Risks

This study indicated significant differences regarding basic attributes, age, age grouping, gender, education, fall history, use or nonuse of long-term medication, the number of medicines used on a long-term basis, and fall risk, demonstrating a significant correlation of the seven basic attributes with fall risk. Further analysis demonstrated that community-dwelling older adults with high fall risk were advanced in age (aged 75 or older), women who had achieved a maximum education level of elementary school graduation, had a history of falls, used medication on a long-term basis, and used a greater number of medicines on a long-term basis. The results of this study in relation to age and age grouping were close to those of Lin and Wang [46] on prevention of and risk factors for falls among community-dwelling older adults, in which age was found to be a risk factor for falls among older adults. Similarly, Kwan et al. [45] indicated that the incidence of falls increased with age and that the annual incidence of falls among community-dwelling adults aged 65 years or older was 28%–35%, whereas the annual incidence rose to 32%–42% among those aged 75 years or older. Chang et al. [47] indicated that gender was a factor greatly affecting falls, and recurrent falls were particularly common among women, which agrees with the results of this study. However, other research [36] contradicted this study in suggesting that gender was not an important factor for falls. This was potentially because women accounted for 66%of this study's participants, and the women in the aforementioned study accounted for less than 50% of the participants. The results of this study suggested that lower education levels are associated with higher fall risks. Similar results came from research by domestic scholars [48] on community-dwelling older adults with chronic diseases and research by Yang et al. [49] on risk factors for falls among older adults in Taiwan: both of these studies suggested that lower education levels were associated with a higher incidence of falls. Domestic scholars [50] also indicated the correlation between education level and falls through a systematic literature review. The investigation by domestic scholars [46] on the prevention of and risk factors for falls among community-dwelling older adults found, similarly, that prior falls were a personal attribute constituting a risk factor for falls among older adults. Another piece of research on the literature [51] indicated that a history of falls was an intrinsic risk factor for falls among community-dwelling older adults. Another such result came from the literature research by Chen et al. [50] indicating that among the biological risk factors causing older adults to fall at home, older adults with prior falls were at higher risk of falling than those with no prior falls. This study showed that study participants engaged the use of more medications and on a longer-term basis exhibited higher risk of falls, a result reflected in several other studies. A systematic literature review by Kwan et al. [45] found the use of multiple medications to be a major risk factor for falls among older adults in Asia. Another such result came from an analysis by domestic scholars [50] of risk factors related to falls among older adults at home. This linked long-term medication use and use of more long-term medicines with higher risk of falling. Moreover, research by domestic scholars [52] indicated that dosage changes and multiple medications increased fall risks. The longitudinal study by Lin et al. [53] also found long-term medication to be a risk factor for falls.

This study demonstrated that the left and right handgrip strengths, the overall grip strength, and results of the TUG test and the 30-second chair stand test significantly affected fall risk, exhibiting a significant correlation of the five physical fitness with fall risk. Further analysis indicated that individuals subject to such risks had weaker left hand, right hand, and overall grip strength; took longer to finish the TUG test; and succeeded in standing fewer times during the 30-second chair stand test. Lin et al. [54] tested community-dwelling older adults' physical fitness and discovered the grip strength test to be a predictor of falls and adverse health conditions among elderly persons. Other research [55, 56] has found grip strength to exhibit a significant positive correlation with fall risk, and a study by Chang et al. [57] on community-dwelling older adults' physical mobility found that declining mobility—grip strength, for example—was a factor that inevitably increased the incidence of falls. The results of this study agreed with those of several others, such as Chin et al. [40], that demonstrated inferior TUG test results to be a major factor affecting falls. In a study of relationships between frailty indexes and adverse health outcomes by Tseng [17], 341 community-dwelling adults aged 65 years or older in Greater Taipei, Linkou, and Taoyuan participated in the “Physical Fitness Tests in the Elderly” retrospective longitudinal study from 2007 to 2009: This showed the incidence of falls in two years to be 2.77 times higher among people who walked slowly than among those who walked at normal speeds (OR =2.77, 95% CI =1.20–6.41, *p* = 0.018), which reflected this study's results. A study by one domestic scholar [34] demonstrated that individuals with prior falls spent more seconds completing the TUG test than did those with no prior falls. Stenhagen et al. [58] found that community-dwelling older adults with higher fall risk also exhibited inferior dynamic balance. This was reflected in the fact that the fall risk became 1.8 times higher for those walking at slower speeds. Inferior dynamic balance ability increased the incidence of falls: The study demonstrated that the fall risk for those who spent longer time completing the TUG test was 1.03–21.4 times greater than the risk for those who spent shorter time completing the test [25, 59, 60]. The aforementioned results were all close to those of this study. The results of this study demonstrated that participants at risk of falls completed fewer stands in the 30-second chair stand test, a result similar to that in Teng [61], indicating that the fall risk of those who achieved 7 or less stands in30-second chair stand test was 5.89 times greater than the risk for those who achieved fewer than 12 stands, and the fall risk for participants who achieved 8 to 11 stands was 2.86 times greater than the risk for those who achieved 12 or more stands. The research of domestic scholars [40] demonstrated inferior 30-second chair stand test results to be a major factor affecting falls, corresponding with results from the study by [34] indicating that study participants with prior falls completed fewer stands in the same test. Based on the results of this study, Dent et al. [62] recommended that frailty should include a multicomponent physical activity program with a resistance-based training component and people with frailty should have received social support as needed for adherence to a comprehensive care plan.

Significant differences were observed in this study for fall risk among older adults at different frailty stages. Further analysis indicated that 30% of the nonfrail older adults were at risk of falls, whereas 50% of the prefrail older adults were at risk, demonstrating a higher proportion of risk in prefrail older adults than in nonfrail ones, a result corresponding with several other ‘studies'. For example, Lu et al. [16] used Fried frailty indexes and cross-sectional research on 189 adults aged 65 years or older in domestic outpatient clinics for chronic diseases and found frailty prediction to be a relevant factor for falls in the preceding year. Similar results came from the study by Tseng [17]of relationships between frailty indexes and adverse health outcomes for 341 community-dwelling adults aged 65 years or older in Greater Taipei, Linkou, and Taoyuan who participated in the “Physical Fitness Tests in the Elderly” retrospective longitudinal study from 2007 to 2009.This study demonstrated that the incidence of falls increased with the frailty level: A statistically significant difference (p = .035) was observed between the nonfrail group's fall rate(24.6%) and the prefrail group's rate(25.7%) [63, 64]. The investigation by a domestic scholar [44] on the prefrailty prevalence among older adults in Taiwan and related factors observed a significant positive correlation between falling history and prefrailty and showed that older adults with prior falls were at higher risk of falling than those without prior falls (OR = 1.80, 95% CI = 1.37–2.36, *p* < 0.0001). These results reflected this study's.

### 5.3. Older Adults' Basic Characteristic, Physical Fitness, and Frailty Stages in Relation to Risk Predictability

This study indicated that community-dwelling older adults with long-term medication exhibited lower fall risks. Regarding long-term medication in risk predictability, the study demonstrated that such adults with long-term medication had a lower fall risk. These results were different to those of several other studies. Hung and Lee [52], Lin et al. [53], and Chen et al. [50] demonstrated that dosage changes and multiple medications often increased older adults' fall risk at home. Systematic literature review by other scholars [45] retrospectively assessed older Asian adults' fall risk factors. Our results showed that participants on long-term medication were subject to low fall risk. The possible reason is that the blood pressure and physical conditions of older people are more stable due to long-term medication; therefore, the risk of falling is reduced.

This study demonstrated that greater grip strength among community-dwelling older adults was associated with lower fall risk. The result resembled that of Wang et al. [63] on the connection between factors related to falls among older adults and bone strength, which indicated that grip strength predicted older adults' physical status and fall risk. Wang [64],on how adding vitamin D and calcium to the diet affected the fall incidence in older women, yielded similar results, demonstrating that fall risk in older adults could be predicted using grip strength. Lin et al. [54] on the physical fitness of community-dwelling older adults showed that future falls and adverse health conditions in older adults could be predicted through grip strength tests. Similarly, significant positive correlations between grip strength and fall risk have been observed in other studies [55, 56]. Chang et al. [57] on community older adults' physical mobility showed that the decline in such physical mobility, such as for grip strength, was a certain cause of falls, and their findings corresponded with this study.

## 6. Limitations

This study had several limitations. First, in relation to the method, the results through cross-sectional research design could represent the physical fitness of community-dwelling older adults during a short period. This indicated only the correlation between the variables and falls and neglected to investigate how factors such as physical fitness and frailty stages in these adults affected falls at various times and in various periods. This limited the inferential levels of the research results. Second, the applied purposive sampling meant that the research results would apply to only a limited range of individuals. Despite the aforementioned limitations, this study had the advantage of being the first comparative study to address basic attributes, physical fitness, and frailty stages in relation to fall risk in community-dwelling older adults in Taiwan. Therefore, the results permit the assessment of frailty and fall risk in the risk group and the development of appropriate care interventions for preventing future falls.

## 7. Conclusion

This study investigated the basic attributes, physical fitness, and frailty stages in relation to fall risk in community-dwelling older adults. Older females with low education, history of falls, weaker overall grip strength; taking longer to finish the TUG test; and standing fewer times during the 30-second chair stand test were at risk of fall. According to the research results, nursing personnel must develop care programs and improve older adults' risk of falls. In the prediction, older people using long-term medication were at lower risk of falling, and the greater the hand grip strength was, the lower the fall risk was. Therefore, comprehensive care plans including multicomponent physical activity programs were necessary.

## Figures and Tables

**Table 1:** Status of community-dwelling older adults in terms of basic characteristic, physical fitness, frailty stage, and fall risk (*n* = 221).

Variable	*n*	(%)	Mean	SD
Age			74.95	6.81
Age group				
65–75 years	117	52.9		
75 years and above	104	47.1		
Sex				
Male	75	33.9		
Female	146	66.1		
Marital status				
Single	54	24.4		
Married	167	75.6		
Living status				
With family	191	86.4		
Solitary	30	13.6		
Educational level				
Illiterate	61	27.6		
Elementary school	121	54.8		
Junior high school and above	39	17.6		
Alcohol consumption history				
No	165	74.7		
Quit	21	9.5		
Yes	35	15.8		
Fall history				
No	172	77.8		
Yes	49	22.2
1 time	38	17.2		
2 times	6	2.7		
3 times	5	2.3		
Chronic disease history				
Osteoarthritis	128	21.9		
Hypertension	117	20.0		
Diabetes	40	6.8		
Myocardial infarction	0	0		
Congestive heart failure	70	12.0		
Hyperlipidemia	90	15.4		
Stroke	12	2.1		
Kidney failure	1	0.2		
Mental disorder	15	2.6		
Glaucoma	13	2.2		
Cataract	99	16.9		
Long-term medication consumption				
Yes	36	16.3		
No	185	83.7		
Number of long-term medications			2.11	1.68
Exercise				
No	13	5.9		
< 2 days/week	17	7.7		
> 3 days/week	191	86.4		
Insomnia				
Yes	89	40.3		
No	132	59.7		
Physical fitness				
BMI			25.18	3.46
BMI grouping				
Normal	76	34.4		
Overweight	83	37.6		
Obese	62	28.1		
Grip strength				
Left hand			25.22	8.11
Right hand			26.31	8.66
Total grip strength			51.54	16.41
Timed Up and Go test			8.12	3.04
30-s chair stand test			16.41	5.02
Frailty stage				
None	145	65.6		
Prefrailty	76	34.4		
Fall risk			46.10	6.28
Yes	88	39.8		
No	133	60.2		

**Table 2:** Analysis of variance of the basic characteristic, physical fitness, and fall risk of community-dwelling older adults with pre-frailty (N = 221).

Variable	Fall risk		*χ* ^2^/t	*p*
	No (*n* = 133)	Yes (*n* = 88)		
Age^b^	72.47 ± 6.06	78.69 ± 6.17	-7.42	≤0.001^∗∗∗^
Age group^a^			45.82	≤0.001^∗∗∗^
65–75 years	95	22		
75 years and above	38	66		
Sex^a^			3.96	.04^∗^
Male	52	23		
Female	81	65		
Marital status^a^			3.09	.07
Single	27	27		
Married	106	61		
Living status^a^			.17	.67
With family	116	75		
Solitary	17	13		
Educational level^a^			32.28	≤0.001^∗∗∗^
Illiterate	20	41		
Elementary school	79	42		
Above junior high school	34	5		
Alcohol consumption history^a^			1.24	.53
No	97	68		
Quit	12	9		
Yes	24	11		
Fall history^a^			8.95	.03∗
No	105	67		
1 time	23	15		
2 times	5	1		
3 times	0	5		
Chronic disease history^a^			13.12	.15
Osteoarthritis	64	64		
Hypertension	64	53		
Diabetes	15	25		
Myocardial infarction				
Congestive heart failure	34	36		
Hyperlipidemia	51	39		
Stroke	6	6		
Kidney failure	1	0		
Mental disorder	9	6		
Glaucoma	9	4		
Cataract	52	47		
Long-term medication consumption^a^			14.79	≤0.001∗∗∗
Yes	101	84		
No	32	4		
Number of long-term medications^b^	1.75	2.65	-3.99	≤0.001^∗∗∗^
Exercise^a^			.17	.91
No	8	5		
< 2 days/week	11	6		
> 3 days/week	114	77		
Insomnia^a^			1.63	.20
Yes	49	40		
No	84	48		
BMI^b^	24.95 ± 3.32	25.51 ± 3.66	-1.17	.24
BMI grouping^a^			3.18	.20
Normal	46	30		
Overweight	55	28		
Obese	32	30		
Grip strength^b^				
Left hand	27.27 ± 8.34	22.13 ± 6.69	5.05	≤0.001^∗∗∗^
Right hand	28.83 ± 8.95	22.50 ± 6.61	6.03	≤0.001^∗∗∗^
Total grip strength	56.11 ± 16.92	44.64 ± 12.91	5.70	≤0.00^∗∗∗^
Timed Up and Go test^b^	7.07 ± 2.00	9.71 ± 3.61	-6.25	≤0.001^∗∗∗^
30-s chair stand test^b^	18.12 ± 5.02	13.84 ± 3.79	7.19	≤0.001∗∗∗
Frailty stage^a^			9.64	.002^∗∗^
No	98	47		
Prefrailty	35	41		

^a^is a categorical variable expressed as *n* (%) and analysed using a chi-square test (*χ*^2^); ^b^is a continuous variable expressed by Mean ± SD and tested using an independent sample *t*-test. ^∗^*p* < .05, ^∗∗^*p* < .01, ^∗∗∗^*p* < .001.

**Table 3:** Fall risk predictability of community-dwelling older adults in basic characteristic and physical fitness.

Independent variable	*B*	SE	*p*	OR	95% CI of OR
Age	.04	.05	.45	1.04	.93-1.16
65–75 years (75 years and above as *ref*)	-.66	.70	.34	.51	.12-2.03
Male (female as *ref*)	.30	.65	.64	1.35	.37-4.87
Educational level (junior high school and above as *ref*)			.31		
Illiterate	.87	.76	.25	2.39	.53-10.67
Elementary school	.22	.66	.74	1.24	.33-4.58
Fall history (3 times as *ref*)			.16		
No	-19.63	16698.96	.99	.000	.000
1 time	-20.72	16698.96	.99	.000	.000
2 times	-20.89	16698.96	.99	.000	.000
Without long-term medications (with long-term medications as *ref*)	-2.08	.82	.01	.12	.02-.62
Number of long-term medications	.13	.12	.29	1.13	.89-1.45
Left hand grip strength	.06	.05	.27	1.06	.95-1.19
Right hand grip strength	-.14	.06	.01	.86	.76-.97
Timed Up and Go test	.12	.09	.21	1.12	.93- 1.37
30-s chair stand test	-.10	.05	.07	.90	.80- 1.01
No frailty (prefrailty as ref)	-.20	.40	.61	.81	.36-1.81

^∗^ *p* < .05, ^∗∗^*p* < .01, ^∗∗∗^*p* < .001.

## Data Availability

Raw data of the current study can be provided upon request.
